# Genome-Wide Association Studies Reveal Susceptibility Loci for Digital Dermatitis in Holstein Cattle

**DOI:** 10.3390/ani10112009

**Published:** 2020-10-31

**Authors:** Ellen Lai, Alexa L. Danner, Thomas R. Famula, Anita M. Oberbauer

**Affiliations:** Department of Animal Science, University of California, Davis, CA 95616, USA; elai@ucdavis.edu (E.L.); aldanner@ucdavis.edu (A.L.D.); trfamula@ucdavis.edu (T.R.F.)

**Keywords:** digital dermatitis, foot warts, lameness, genome-wide association study, linear mixed model, random forest, Bayesian estimation, sustainability, animal welfare

## Abstract

**Simple Summary:**

Foot warts (FW), a leading cause of foot problems in dairy cattle, is a welfare concern and causes financial losses due to treatment and reduced milk production. Foot warts, or the technically correct term of digital dermatitis, result from a bacterial infection followed by delayed healing due to both genetic and environmental factors. Dairy farmers are already combatting FW through environmental control, but they do not have genetic selection tools because the genetics influencing FW susceptibility are largely unknown. We sought to identify the genetics associated with FW which can be incorporated into genetic selection tools. Farmers can then use these genetic selection tools to breed cows that are less susceptible to FW. We identified promising genes that play a role in the immune response and wound healing—immune functions that, if impaired, could increase a cow’s susceptibility to FW. Though these genes were promising, their associated genetic markers had very little influence on FW susceptibility when compared to environmental management. Thus, the findings imply that the best approach for reducing FW prevalence is likely through combining a genetics approach with environmental management.

**Abstract:**

Digital dermatitis (DD) causes lameness in dairy cattle. To detect the quantitative trait loci (QTL) associated with DD, genome-wide association studies (GWAS) were performed using high-density single nucleotide polymorphism (SNP) genotypes and binary case/control, quantitative (average number of FW per hoof trimming record) and recurrent (cases with ≥2 DD episodes vs. controls) phenotypes from cows across four dairies (controls *n* = 129 vs. FW *n* = 85). Linear mixed model (LMM) and random forest (RF) approaches identified the top SNPs, which were used as predictors in Bayesian regression models to assess the SNP predictive value. The LMM and RF analyses identified QTL regions containing candidate genes on *Bos taurus* autosome (BTA) 2 for the binary and recurrent phenotypes and BTA7 and 20 for the quantitative phenotype that related to epidermal integrity, immune function, and wound healing. Although larger sample sizes are necessary to reaffirm these small effect loci amidst a strong environmental effect, the sample cohort used in this study was sufficient for estimating SNP effects with a high predictive value.

## 1. Introduction

Lameness, or abnormal gait, affects 16% of dairy cows in the United States, making lameness the second most prevalent disease in dairy cattle after mastitis [1]. Digital dermatitis (DD) is a common cause of lameness, comprising 70.9% and 36.0% of lameness cases in heifers and cows, respectively [1]. The economic impacts of DD are $64 to $153 per episode due to reduced milk production, discarded milk, treatment costs, and additional labor [2,3]. Furthermore, premature culling obligates producers to expand their replacement heifer herd. Because heifers consume inputs without contributing to milk production, a larger replacement heifer herd inflates the economic cost [4] and carbon footprint [5] per unit of milk. Thus, reducing the incidence of DD and associated lameness has great potential to benefit animal welfare, the producer’s profit margin, and the environment, bolstering the three pillars of sustainability.

Heritability estimates for DD range from 0.01 to 0.4 [6,7,8], indicating genetic contributions to DD susceptibility along with a strong environmental influence. Reducing DD incidence, therefore, will likely be achieved through a combination of management and genetic approaches informed by the etiology of DD. Although the etiology of DD has not been completely elucidated, multiple bacterial phylotypes belonging to the genus *Treponema* are consistently found in DD lesions [9]. Accordingly, the main environmental management method for reducing DD incidence is medicated foot baths [10,11], though this treatment is expensive, with estimates of ~$42 per cow per year [12]. Additionally, the disinfectant compounds commonly used in foot baths raise environmental and health concerns, as the primary ingredients, copper sulfate and formaldehyde, are environmental pollutants [13] and carcinogenic [14], respectively. To alleviate these issues and improve DD prevention, some producers emphasize feet and leg conformation scores or indices that include claw health when selecting sires and, increasingly, rely upon genetic testing for heifers. However, the low genetic correlation between conformation traits and claw health impairs efficient indirect selection against claw lesions when using selection on conformation traits [15]. 

Currently, no selection index exists specifically for DD susceptibility. Targeted genetic selection against DD and associated lameness requires the identification of genomic regions influencing DD susceptibility. To find the contributing quantitative trait loci (QTL), genome-wide association studies (GWAS) have been undertaken, although the results to date have been discordant. Previous studies found significant and suggestive single nucleotide polymorphisms (SNPs) on *Bos taurus* autosomes (BTAs) 1, 3, 5, 6, 8, 9, 10, 14, and 26 [6,16,17,18,19], or no suggestive or significant SNPs [20]. 

In an effort to improve upon and refine past studies, the present study used strict phenotyping, dairies with similar management practices, and a high-density SNP genotyping array to identify the associations between DD and genomic regions. We hypothesized that certain genetic markers would be associated with DD susceptibility, and that those markers would have small effects. Our results revealed multiple small-effect SNPs were associated with DD and defined QTL that contained candidate genes related to immune function and wound healing, supporting our hypothesis.

## 2. Materials and Methods

Four commercial dairies in the Central Valley of California, two of which had participated in our previous heritability study [8], provided hoof trimming records and blood samples from which to generate genotypic data. All the procedures were conducted in accordance with the ethical standards set by the University of California, Davis, and approved by the Institutional Animal Care and Use Committee.

### 2.1. Phenotypic Data

Hoof trimming records were used to generate binary and quantitative phenotypes. Claw lesions and lameness issues were diagnosed by a single hoof trimmer servicing three of the dairies (dairies A, B, and C), and a different hoof trimmer servicing the fourth dairy (dairy D). The hoof trimmer servicing three dairies was trained by Dr. Steven Berry, a veterinarian specializing in claw lesions who offered hoof trimming training workshops to the industry and was a coauthor of our earlier paper [8], and the other trimmer shadowed trained trimmers to standardize the diagnostics. Claw lesions were diagnosed and recorded while the cow was restrained. Claw lesion types and the foot with DD lesions were recorded into the cow’s electronic record (dairies A and D) or maintained in a hard copy format (dairies B and C). Each type of claw lesion (e.g., DD, sole ulcer, laminitis, white line disease, foot rot, etc.) and miscellaneous lameness event (e.g., rock, cut, etc.) was tallied for each cow.

Both hoof trimmers utilized similar criteria for defining instances of DD in the cattle to reduce the phenotypic classification variability; specifically, DD was recorded for cows with raised, inflamed lesions on the skin above the heel of the foot or above the interdigital space on the front of the foot. Routine hoof trimming varied by dairy: cows were trimmed at the beginning and middle of lactation, during dry off, and when exhibiting altered gait (dairy A); during dry off and when exhibiting altered gait (dairy B and C); and only when exhibiting altered gait (dairy D). Cases were defined as cows who had exhibited at least one DD lesion, whereas controls had no DD or other lameness records and were 6.5 years of age or older to avoid misphenotyping younger cows who had insufficient time to develop lameness events. Cows may have multiple bouts of DD over their lifetime and, in some cases, the first instance of DD may have occurred before the cows were in milk (i.e., as heifers). Because DD lesions typically last for four to six months [21,22], we defined independent DD lesion episodes as those that were separated by at least six months. Cows with two or more independent DD episodes were considered recurrent cases. Digital dermatitis records that were less than six months apart were considered repeated records of one persistent DD episode. Digital dermatitis was analyzed as a binary phenotype to identify loci influencing general susceptibility to DD and as a quantitative phenotype calculated as the total number of independent DD lesions a cow had divided by the total number of hoof trimming records to standardize the number of lesions by the number of hoof trimming records for each cow. Consequently, the quantitative phenotype for a control cow was zero. Digital dermatitis was also analyzed as a recurrent phenotype (cases with ≥2 DD episodes vs. controls) to identify the loci contributing to reoccurring DD episodes.

### 2.2. Genome-Wide Association and Linear Mixed Model Analyses

Genomic DNA was extracted from whole blood samples using the QIAGEN QIAamp DNA Blood Mini Kit (QIAGEN Inc., Valencia, CA, USA) and quantified using the NanoDrop (ND-2000 v3.2.1) spectrophotometer (Thermo Scientific, Wilmington, DE, USA). DNA samples were genotyped on the BovineHD BeadChip (777962 SNPs, Illumina Inc., San Diego, CA, USA) by GeneSeek (Lincoln, NE, USA). Raw and processed microarray data were submitted to the NCBI Gene Expression Omnibus database (GEO series record GSE159157). Illumina’s GenCall algorithm was used to call genotypes.

GWAS were performed using the binary, quantitative, and recurrent phenotypes using the SNP coordinates from the ARS-UCD1.2 map (accessed August 2020 from the National Animal Genome Research Project’s Cattle Genome Analysis Data Repository (https://www.animalgenome.org/repository/cattle/UMC_bovine_coordinates/), version last modified 11 September 2018). The quality filtering of SNP genotypes was performed using PLINK 1.9 [23,24] to remove from further analysis any cows having less than 5% of all SNPs genotyped and SNPs missing genotypes in more than 5% of the cows. SNPs with a minor allele frequency of less than 0.05 were removed to exclude rare variants, and SNPs that deviated significantly from the Hardy–Weinberg equilibrium (*p* < 1 × 10^−6^) in controls were removed to exclude systematic genotyping errors.

Family structure is extremely prevalent in the dairy population from breeding elite bulls to hundreds to tens of thousands of cows. To visualize the genetic similarity among cows at this initial dairy, a multi-dimensional scaling (MDS) analysis was performed and the first two dimensions were plotted. The GWA analyses were performed using the genetic analysis program Genome-wide Complex Trait Analysis (GCTA) [25] to fit a linear mixed model (LMM) that tests for the association of SNP genotypes with binary and quantitative DD phenotypes. An LMM was selected for its ability to incorporate a genetic relatedness matrix to correct for familial relatedness and population structure. Linear mixed models are designed for quantitative phenotypes, as LMMs assume that phenotypes are normally distributed; however, LMMs have also been routinely used to analyze binary traits [26,27]. A genetic relatedness matrix was computed and included along with farm as a covariate in the LMM. When fitting the LMM for each SNP, the LMM included the chromosome of the candidate SNP being tested. To reduce false positive associations due to multiple testing across many loci without being overly stringent, the effective number of independent SNPs (M_e_) after linkage disequilibrium (LD) pruning was determined using the Genetic Type I error calculator (GEC) and used as the denominator for Bonferroni-corrected thresholds [28]. Significant SNPs were defined as those with *p* < 0.05/M_e_, whereas suggestive SNPs were defined as having *p* < 1/M_e_ [29]. To calculate the genomic inflation factors (λ_GC_), chi-squared test statistics were first generated from association *p*-values, and the median of the resulting chi-squared distribution was divided by the median of the expected chi-squared distribution. Quantile-quantile plots (qqplots) and Manhattan plots were plotted in R [30] using the package qqman [31].

### 2.3. Random Forest Analysis

Random forest (RF) analysis was performed as an additional method for identifying SNPs that appeared to importantly contribute to disease phenotypes. Random forests do not make any assumptions about the inheritance model (additive, dominant, recessive) and are able to test multiple SNPs jointly for association with phenotype. Additionally, the RF approach is unaffected by an uneven farm distribution of cases and controls because RF builds decision trees and estimates the importance of each feature by the frequency it appears in the decision trees, rather than estimating parameters for a model. Consequently, RFs avoid estimating parameters for which there are no data. These properties make RFs well equipped to identify structure within complex genetic architectures like DD susceptibility. Specifically, RF can accommodate data despite uneven sampling across farms, in which contributing SNPs may have different modes of inheritance and where epistasis is likely prevalent. 

After converting quality-filtered binary PLINK files into VCF files split by chromosome in PLINK 1.9 [24,32], all the missing genotypes were imputed using BEAGLE 5.1 [33] because the RF analysis cannot handle missing genotypes. The resulting VCF files were converted back to binary PLINK files, which were LD-pruned using a threshold of R^2^ ≥ 0.90 to avoid diluting the importance of SNPs in strong LD during the RF analysis [34] and recoded to additive and dominant component files suitable for importing into R. The additive component (i.e., genotypes coded as 0/1/2 minor alleles) was used as input for the RF analysis in R using the caret package [30,35]. For binary and quantitative phenotypes, RF analysis was implemented with all genome-wide SNPs in one run to estimate the relative importance of explainers, comprised of SNP genotypes and farm. For both runs, the same random sample of two thirds of the cows was used to train the model and calculate variables of importance for each explainer. The RF run for each phenotype built 500 decision trees that included three values of *mtry*, the number of predictors considered at each node of the tree. The value of *mtry* that yielded the most accurate model was used as the final model. The most important explainer was assigned an importance variable of 100, and the other explainers were assigned importance variables relative to the most important explainer (e.g., an explainer with an importance of 50 is 50% as important as the most important explainer). To assess the accuracy of the final model, the remaining third of cows was used as the test population, using the explainers and their relative importance to predict phenotype.

After evaluating the model accuracy using the test population, a threshold of importance was determined by ranking and plotting the SNPs the RF identified as important for each chromosome in a scree plot and finding the rank of the second-order point of inflection using the d2uik option in the inflection package in R [36,37]. SNPs ranking equally as or more important than this threshold were considered important and included in further analyses.

### 2.4. Bayesian Regression to Assess Model Predictability and Validation

To assess the collective predictive ability of the top SNPs identified in the LMM and RF analyses, the top SNPs from each analysis (i.e., significant and suggestive SNPs from LMM analyses, important SNPs from RF analyses) were tested for association with phenotype using Bayesian regression. Bayesian regression was selected because of its ability to fit multiple SNPs simultaneously while also recognizing that the majority of SNPs have small effects on DD susceptibility [20,38], that some SNPs are likely correlated due to LD, and that not all farms contributed controls to the analyses. Additionally, Bayesian regression enables the thorough evaluation of model fit through leave-one-out (LOO) validation and posterior predictive checking (PPC), the latter of which is a uniquely Bayesian feature.

Suggestive and significant SNPs from the LMM GWAS and important SNPs from the RF analysis were used as predictors along with farm in each Bayesian regression model. Similar to the RF analyses, SNP genotypes were coded as 0/1/2 minor alleles. A Bayesian regression model was fitted for each combination of GWAS method (LMM and RF) and phenotype (binary and quantitative), such that four models were fitted: LMM-binary and RF-binary were fitted using a Bayesian logistic regression model, and LMM-quantitative and RF-quantitative were fitted using a Bayesian generalized linear model for continuous data. Susceptibility to DD appears to be complex and the majority of SNP effects are likely to be small [20,38]. To reflect this distribution of SNP effects, a normal prior with a small-scale N (0,1) was used for the distribution of predictors for all four models. Each of the four models was fitted by sampling from the posterior distribution using the Hamiltonian Monte Carlo algorithm, a Markov chain Monte Carlo (MCMC) algorithm, using the rstanarm package in R [39]. Four parallel chains sampled the posterior distribution, and each chain was run for 10,000 iterations with a warmup of 2500 iterations, keeping every 25th iteration to avoid autocorrelation. 

Unlike frequentist regression, which would output a point estimate of each SNP effect, Bayesian regression outputted a distribution of where the true value of each SNP effects fell, defined by the Bayesian uncertainty interval (UI). SNPs with 95% UIs that did not include zero were considered significantly associated with DD susceptibility. For each significant SNP, the probability of disease given a genotype at the significant SNP (coded as 0/1/2 minor alleles) and a 0 genotype at all other SNPs was calculated using the median of SNP effect estimates as point estimates in the inverse logit equation using the R package arm [40]. Diagnostic and Bayesian UI plots for the posterior medians of SNP effects were plotted using the bayesplot package. Leave one out cross validation was performed using the loo package [41,42] in R to predict the phenotype of each cow using the SNP effects estimated from all other cows. The reliability of prediction was assessed using the Pareto *k* diagnostic values outputted from the LOO analysis. Posterior predictive checking (PPC) from the bayesplot package [43] was used to assess the goodness of fit of the model. Posterior predictive checking assessed how well the estimated predictor effects were able to simulate phenotypes with a similar distribution to that of the observed phenotypes.

### 2.5. Defining and Annotating QTL Regions

For the significant and suggestive SNPs identified in the LMM analyses and the important SNPs identified in the RF analyses, the QTL boundaries and regions were defined and annotated. Because SNPs are more likely to be in LD with causal variants than be causal themselves, the linkage disequilibrium in the regions flanking these top SNPs was used to define the boundaries of QTL, per the methods used in previous GWAS studies [44,45]. Specifically, SNPs within 5 Mb of significant and suggestive SNPs that were also in LD (r^2^ ≥ 0.5) were considered as belonging to the same QTL. The SNPs furthest upstream and downstream that were in LD with the target suggestive or significant SNP defined the boundaries of the QTL. Overlapping QTL were combined into one QTL. QTL from the LMM and RF analyses were compared to discern whether the two analyses found the same QTL. QTL regions that were identified in both LMM and RF analyses were explored for candidate genes. Additionally, QTL defined by SNPs that were significant in the Bayesian regression analyses were also explored for candidate genes. Candidate genes were defined as genes falling in QTL regions identified in both LMM and RF analyses or in QTL defined by SNPs that were significant in Bayesian regression and were functionally relevant to DD etiology.

To annotate the QTL regions, the genomic regions search in FAANGMine v1.1 [46] using the ARS_UCD1.2 assembly was implemented to find genes within the QTL regions. The RefSeq identifiers of genes within the QTL were used in a gene ontology and pathway enrichment analysis in FAANGMine to discern whether the genes belonged to higher-order functions and pathways related to DD etiology. For the gene ontology and pathway enrichment analyses, the Benjamini Hochberg test correction was used to correct for multiple testing, and all the RefSeq genes in *B. taurus* were used as the background population. To identify the functions of individual genes, protein coding genes in QTL defined by SNPs that were significant in two analyses (i.e., LMM, RF, and/or Bayesian regression) were searched in the Mouse Genome Informatics batch query database (http://www.informatics.jax.org/batch) using the mammalian phenotype option [47].

## 3. Results

### 3.1. Descriptive Data

Hoof trimming records for 1382 DD-affected cows at dairies A, B, and D from 2002 to 2019 were used to calculate the age of onset statistics. Dairy C did not have hoof trimming records from the beginning of the cows’ lives and was thus excluded from calculating the age of onset statistics. The average age of onset for the first episode of DD observed was 3.7 (SD = 1.6) years old and the median was 3.5 years old, indicating a minimum age of 6.5 years old for controls was sufficiently stringent to avoid misphenotyping younger cows. The cases and controls were sampled from 2013 to 2020. Cases were sampled from all four dairies, whereas only dairies A and D had control cows that met our stringent age and soundness requirements (Table 1). In total, 222 cows were genotyped (cases n = 90, controls n = 132), of which six were removed during quality filtering (cases n = 3, controls n = 3), leaving 216 cows for analysis (cases n = 87, controls n = 129). Of the 87 cases, 24 had recurrent FW episodes and were used in the GWAS of controls vs. recurrent FW cases. Forty-seven percent of the DD cases no other claw lesions other than DD during their lifetime. The remaining cases had, in addition to clearly identifiable DD, abscesses, sole fracture, sole ulcers, or bruising. One cow also had foot rot in addition to DD. Of these other claw lesions, only foot rot was considered infectious, whereas the other concomitant lesions were noninfectious and associated with excessive wear of the claw due to hard flooring and/or metabolic issues [48].

**Figure 1:**
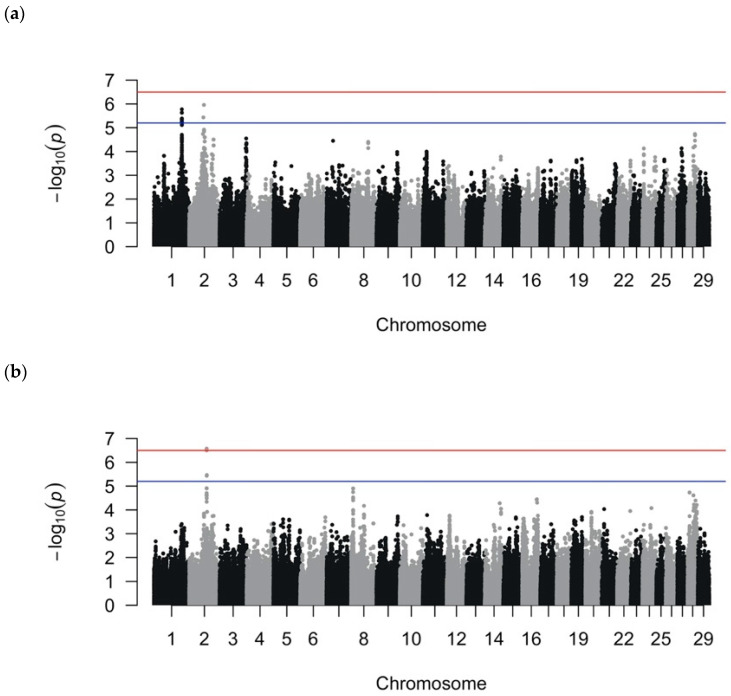
Manhattan plots from the linear mixed model genome-wide association analyses using (**a**) binary phenotypes designating the presence of digital dermatitis (DD) lesions or the absence of any lameness issues and (**b**) quantitative phenotypes calculated by dividing the number of DD episodes by the total number of hoof trimming records. The red line indicates the threshold for genome-wide significance (Bonferroni-corrected using the number of independent SNPs at *p* < 0.05), and the blue line indicates the threshold for suggestive significance (Bonferroni-corrected using the number of independent SNPs at *p* < 1).

After quality control filtering, 560,277 SNPs remained for the LMM analysis, and 222,060 SNPs (40%) for the RF analyses remained after LD pruning (r^2^ > 0.90). The MDS analysis indicated no obvious population stratification (Figure S1). The effective number of SNPs (i.e., SNPs that were not in LD) was approximately 158,000 SNPs, yielding a cutoff of significance at 3.2 × 10^−7^ or 6.5 on the −log_10_(*p*) scale and a suggestive cutoff at 6.3 × 10^−6^ or 5.2 on the −log_10_(*p*) scale. Manhattan plots for the LMM binary and quantitative analyses are shown in Figure 1 and suggestive and significant SNPs, in Table 2 and Table 3. For the recurrent LMM GWAS, the Manhattan plot is depicted in Figure S2 and suggestive and significant SNPs in Table S1. The genomic inflation factors were 0.97 for the binary and quantitative GWASs and 1.0 for the recurrent GWAS; when considered in conjunction with the qqplots, the analyses sufficiently accounted for population structure (Figure S3). In separate analyses, we removed outlier control cows, defined as having a value < −0.10 in the first coordinate and a value < −0.08 in the second coordinate of the MDS plot, and the conclusions of association remained unchanged (Figure S4). Our method of correction for multiple testing (i.e., using the effective number of independent SNPs as the denominator for Bonferroni correction) resulted in more stringent significance thresholds than those based on false discovery rate that are used in other GWASs for DD [6,16,20].

### 3.2. SNPs Associated with DD as a Binary Phenotype

The binary LMM GWAS detected 22 suggestive SNPs on BTA1 that fell in the last three introns of *SLC9A9* and two suggestive intergenic SNPs on BTA2 (Table 2). When used to define QTL boundaries, the 22 suggestive SNPs on BTA1 were all in LD and defined one 271.2 kb QTL region at BTA1:125550933–125822143 containing three genes: a long-noncoding RNA gene (LOC112447746), a tRNA-CAU gene, and *SLC9A9*. The BTA2:63365256 (BTA-47853-no-rs) SNP on BTA 2 identified a 2.4 Mb QTL region at BTA2:60971364–63389576 containing 25 genes, whereas the other SNP identified on BTA 2, BTA2:65836042 (BovineHD0200019142), was not in LD with neighboring SNPs (r^2^ < 0.5). Because the number of genes discovered from the LMM QTL was limited, no gene ontologies or pathways were overrepresented.

**Figure 2:**
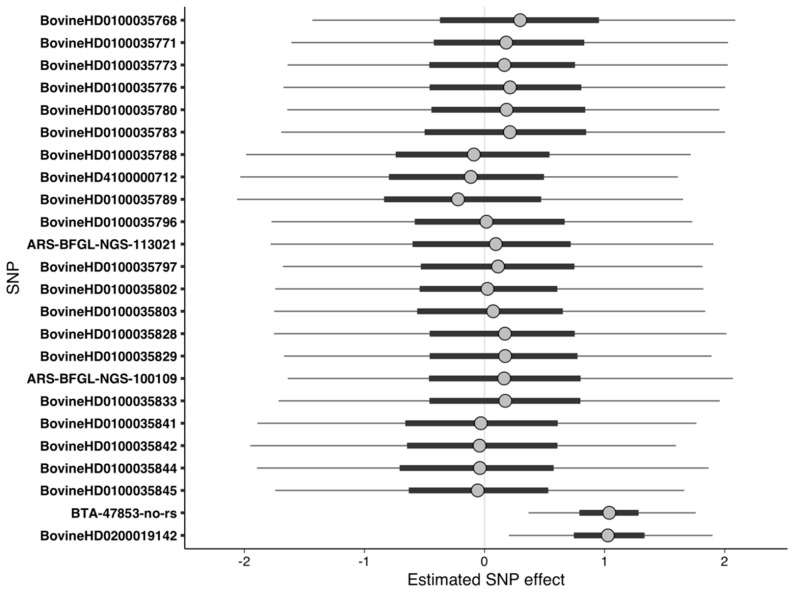
Uncertainty interval (UI) plot for suggestive SNPs on BTA1 (BovineHD0100035768 through BovineHD0100035845) and BTA2 (BTA-47853-no-rs and BovineHD0200019142) from the linear mixed model GWAS using binary phenotypes. Dots represent the median of the SNP effect estimates from Markov chain Monte Carlo draws, thick bars indicate the 50% UI, and the thin lines indicate the 95% UI. SNPs with 95% UI not overlapping zero were considered significant. Positive values of predictor effect estimates indicate a higher risk of DD, whereas negative values indicate a lower risk of DD.

When suggestive SNPs from the LMM-binary GWAS were used as predictors in the Bayesian regression models, MCMC sampling was able to efficiently explore the posterior. Though the effects of SNPs on BTA1 were not significantly different from zero at 50% UI, the effects of the two SNPs on BTA2 (BTA-47853-no-rs and BovineHD0200019142) were significantly different from zero at 95% UI (Figure 2, Table 2). Unlike a frequentist 95% confidence interval, which defines the range within which the true value of the SNP effect falls 95% of the time in repeated sampling, a Bayesian 95% uncertainty interval indicates there is a 95% probability that the true value of the SNP effect falls within the range. For example, to give context for the impact of SNP effect size, each minor allele at BTA-47853-no-rs and BovineHD0200019142, respectively, increased the log odds of having DD by 1.3 and 1.5, using the median as the point estimate for SNP effect. A 1.3 increase in the log odds of having DD for each minor allele at BTA-47853-no-rs corresponded to an increase in the probability of having DD by 22% and 54% for heterozygotes and homozygotes of the minor allele relative to homozygotes of the major allele. A 1.5 increase in the log odds of having DD for each minor allele at BovineHD0200019142 corresponded to a 25% and 60% increase in the probability of having DD for the heterozygotes and homozygotes of the minor allele, relative to the homozygotes of the major allele. The relatively large increases in the probability of having DD from each additional minor allele reflects the high minor allele frequency in cases (45%) relative to controls (25%) in this population. Additionally, the magnitude of increase in the probability of DD also depended upon the genotype of the cow at other SNPs. For instance, a cow with a genotype other than homozygous major for all SNPs could have a smaller increase in the probability of DD with each additional minor alelle at BTA-47853-no-rs or BovineHD0200019142.

Using the LMM-binary suggestive SNPs as predictors in the LOO analysis, Pareto k diagnostic values were acceptable (k ≤ 0.7) for all cows, indicating that the estimated SNP effects were collectively predictive of phenotype within the original population. The LOO analysis indicated that the effective number of predictors in the model was 6.6, considerably lower than the 27 predictors that were actually in the model due to correlated predictors: the SNPs on BTA1 were in LD, and this correlation among predictors reduced the effective number of predictors. The PPC indicated that the observed and simulated data were similar to each other (Figure S5), supporting that the predictor estimates were collectively predictive of phenotype. 

Random forest analysis revealed that farm was ranked as the most important explainer, and consequently the importance of SNPs was expressed as the percentage of importance relative to farm. Of the three values of *mtry* that were tested (6, 666, and 222,061), *mtry* = 666 yielded the most accurate model and was selected for further analyses. The accuracy of the selected model (0.69 with 95% CI 0.57–0.80) was not significantly different from the baseline no information rate (in this case, the proportion of controls: 0.64, *p* = 0.20), indicating that the model was unable to call case and control phenotypes more accurately than simply calling the more common phenotype. Random forest analyses found 26 important SNPs from the RF-binary, and using LD to determine the QTL boundaries defined 23 QTL for the RF-binary dataset (Table 3), one of which was the same QTL on BTA1:125550933–125822143 identified from the LMM-binary GWAS. Within the RF-binary QTL, FAANGMine found 566 genes, of which 129 and 188 were used in the pathway and gene ontology enrichment analysis. The Kyoto Encyclopedia of Genes and Genomes (KEGG) pathway herpes simplex virus 1 infection and the Reactome pathways P2Y receptors and nucleotide-like (purinergic) receptors were significantly enriched (Benjamini Hochberg *p* = 0.003, 0.021, and 0.035, respectively). 

**Figure 3:**
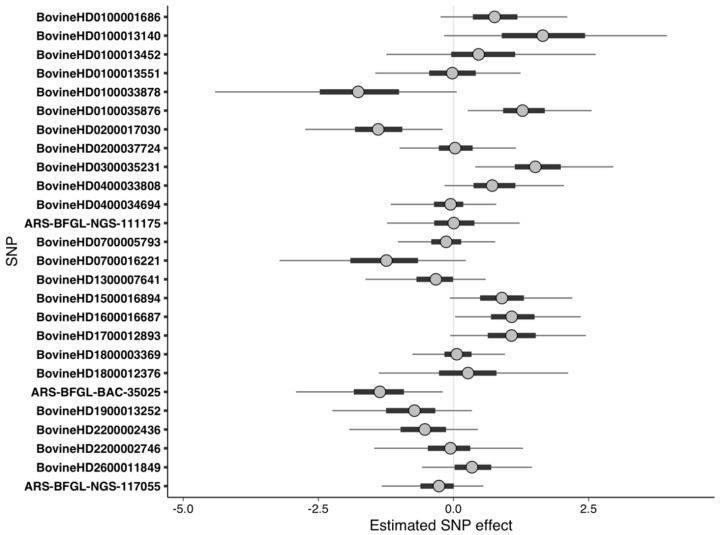
Uncertainty interval (UI) plot for important SNPs from the random forest analysis using binary phenotypes. Dots represent the median of the SNP effect estimates from the Markov chain Monte Carlo draws, thick bars indicate the 50% UI, and the thin lines indicate the 95% UI. SNPs with 95% UI not overlapping zero were considered significant. Positive values of predictor effect estimates indicate a higher risk of DD, whereas negative values indicate a lower risk of DD.

When important SNPs from the RF-binary analyses were used as predictors in the Bayesian logistic regression model, four SNPs had estimated effects that were significantly different from zero, including the SNP defining the QTL at BTA1:125550933–125822143 (Table 3, Figure 3). The important SNPs from the RF-binary analyses were not as predictive of phenotype within the population compared to the suggestive SNPs from the LMM-binary analysis, as evidenced by 13% of cows having high Pareto k diagnostic values (k > 0.7) from the LOO analysis. The lower predictability indicates that the RF was able to find small effect SNPs, but also found some noninformative SNPs. Though the PPC indicated that the observed and simulated data were similar to each other (Figure S6), this similarity was likely due to overfitting.

### 3.3. SNPs Associated with DD as a Quantitative Phenotype

The quantitative LMM GWAS identified seven significant and two suggestive SNPs, all of which were intergenic (Table 4). The gene nearest to these nine SNPs was a suppressor of cytokine-signaling 6-like pseudogene (LOC615204) falling between the seven significant and two suggestive SNPs. When these nine SNPs were used to determine the QTL boundaries, all nine SNPs were in LD (r^2^ > 0.5) and defined a 2 Mb QTL region at BTA2:77930065–79925981 (Table 4). This 2 Mb QTL region included nine genes, including LOC615204. The recurrent DD cases vs. controls placed more emphasis on finding genetic differences between controls and cases with more DD cases, similar to the LMM-quantitative GWAS; however, the LMM GWAS using recurrent DD cases vs. controls identified QTL regions in common with the LMM-binary and not the LMM-quantitative GWAS. In the recurrent GWAS, the same SNPs observed on BTA1 from the LMM-binary analyses formed a peak of association but did not reach suggestive significance, whereas three SNPs on BTA2 in addition to the two detected in the LMM-binary GWAS reached suggestive significance (Figure S2, Table S1). The three additional suggestive SNPs on BTA2 revealed by the recurrent analysis defined a 328 kb QTL at BTA2:65836042–66217730 that was not in LD with the QTL at BTA2:60971364–63389576 defined by BTA-47853-no-rs at BTA2:63365256 in both the binary and recurrent LMM GWASs (Table S1).

When the significant and suggestive SNPs from the LMM-quantitative analysis were used as predictors in Bayesian regression, MCMC sampling to fit the model was unable to efficiently explore the posterior likely because the phenotypes did not follow a normal distribution as expected by the model. This resulted in unreliable results and thereby prevented further analyses. The limited number of genes within the LMM-quantitative QTL on BTA 2:77930065–79925981 prevented the detection of overrepresented gene ontologies or pathways.

Random forest analysis using quantitative phenotypes revealed that, similar to the RF-binary rankings, farm was ranked as the most important explainer. The 15 important SNPs identified from the RF-quantitative analysis defined 13 QTL distinct from those defined in the LMM-quantitative analysis (Table 5). The RF-quantitative QTL contained 124 genes. The 28 and 13 genes that were used in pathway analysis using KEGG and Reactome pathways did not find enriched pathways. The 37 genes used in gene ontology enrichment analysis did not have significantly overrepresented gene ontologies after multiple testing correction.

**Figure 4:**
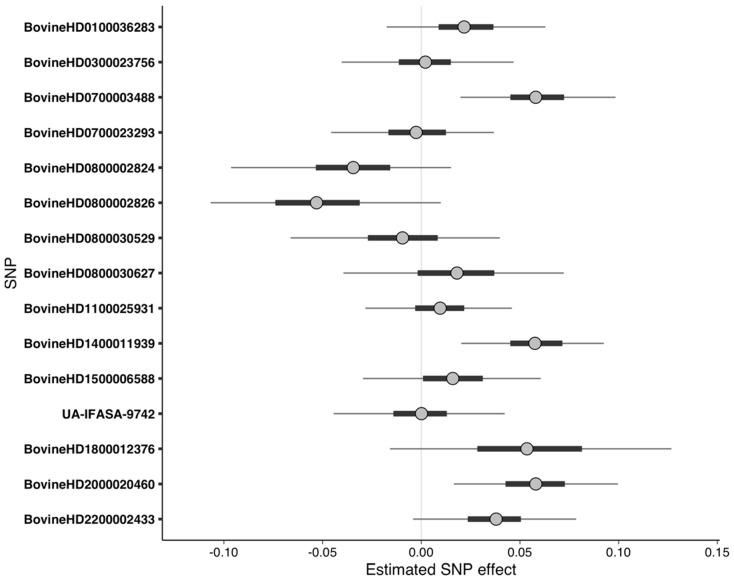
Uncertainty interval (UI) plot for important SNPs from the random forest analysis using quantitative phenotypes. Dots represent the median of the SNP effect estimates from the Markov chain Monte Carlo draws, thick bars indicate the 50% UI, and the thin lines indicate the 95% UI. SNPs with 95% UI not overlapping zero were considered significant. Positive values of the predictor effect estimates indicate a phenotypic value for DD, whereas negative values indicate a phenotypic value for DD.

Although no pathways or gene ontologies were enriched from the RF-quantitative dataset, the important SNPs detected were nonetheless predictive of phenotype when used as predictors in Bayesian regression. MCMC sampling to fit the Bayesian model was able to explore the posterior sufficiently, resulting in convergence. Three of the important SNPs had effect sizes significantly greater than zero at 95% UI (Figure 4, Table 5). The LOO analysis indicated that the 15 SNPs were predictive of quantitative phenotype, as all the cows had Pareto k diagnostic values that were acceptable (k ≤ 0.7). The PPC demonstrated that the simulated data followed a similar distribution to the original data, though the frequency of more extreme phenotypes was dampened (Figure S7).

## 4. Discussion

The genetic component of DD susceptibility is highly complex and heterogeneous [20,38], as demonstrated by the numerous and varied QTL detected in previous studies [6,16,17,18,19,20]. We sought to further identify the QTL contributing to DD susceptibility using a high-density SNP array and LMM and RF analytical approaches on well-phenotyped DD cases and controls. The LMM GWAS and RF analyses revealed suggestive, significant, and important SNPs that defined QTL regions in binary, quantitative, and recurrent DD phenotypes. The LMM GWAS using recurrent DD cases vs. controls indicated that the recurrent DD cases were contributing to the significance of association in the LMM-binary GWAS on BTA1 and BTA2, but not in the LMM-quantitative GWAS. Bayesian regression allowed for an intuitive estimate of SNP effects and the robust evaluation of model fit through the LOO and PPC analyses, providing additional distinctions of informative and noninformative SNPs among the top SNPs. QTL regions were explored for candidate genes if the QTL was defined by the top SNPs (i.e., significant or suggestive SNPs from LMM analyses or important SNPs in RF analyses) that were also significant in Bayesian regression or were top SNPs in both LMM and RF analyses. That is, nine QTL were investigated further (Table 6).

Within these QTL regions, we identified likely candidate genes based on their relevance to DD etiology. DD is associated with Treponema bacteria invading the dermis and epidermis, likely through hair follicles, and results in a raised erosive lesion [21,49]. The infection elicits a strong initial activation of the innate immune response [50] that is then attenuated by the treponemes [51], leading to prolonged inflammation and a delayed adaptive immune response [52]. Within the adaptive immune response, the antibody-mediated immune response is mainly responsible for defending the host against extracellular pathogens [53], such as treponemes. After the immune response, the skin then attempts to heal the wound, a process that is also impaired by treponemes [51]. As such, changes in the sequence or expression of genes related to maintaining epidermal integrity, immune response, or wound repair could affect a cow’s DD susceptibility and the persistence of a DD lesion. Previous work has indicated that genes related to these three functions were dysregulated in DD lesions [18,51]. Therefore, we considered candidate genes as those with associated phenotypes, as determined by MGI, that pertained to these functions and fell within the six QTL regions, resulting in six candidate genes: *CXCR4*, *MGAT5*, *CACNA1A*, *TERT*, *SLC9A3*, and *AHRR* (Table 6, Table S2). All six candidate genes were related to immune function, and *TERT* was also associated with skin hyperplasia and wound healing (Table S2). Similarly, we defined functionally relevant gene ontologies and pathways as those related to these three functions. The QTL on BTA18 contained 16 zinc finger genes that were part of the herpes simplex virus 1 infection pathway, implying an immune function of these genes that could also play a role in DD infection (Table 6).

A limitation of the study is the small sample size. Minimizing phenotypic variation and increasing sample size are both methods to improve the detection of small-effect SNPs, but often pursuing one of these approaches comes at the expense of the other—for example, in this study, large sample size. Our strict phenotypic criteria also caused the controls to be from only two dairies, which was partially accounted for in the LMM analyses by including a covariate term. While the uneven sampling of dairies can be problematic in frequentist methods such as LMM, those issues were avoided in RF analyses and Bayesian estimation because these models account for parameters that did not exist (e.g., a control cow from Farm B or C). Furthermore, the SNPs that defined the QTL regions containing promising candidate genes were significant, suggestive, or important in the LMM and RF analyses, some of which also had nonzero effect sizes estimated from Bayesian regression despite the small sample size. For the quantitative phenotypes, a larger sample size might have more normally distributed phenotypes that the model expects, thereby improving the efficiency of MCMC sampling and more accurate SNP effect estimates. Although the sample size of this study was limited due to our intentionally reducing phenotypic variation, which may have prevented the detection of additional small SNP effects, the sample size was sufficient to very accurately predict the phenotype within the original population. Future replication studies are necessary to determine how well the SNP effects estimated in this study population can be extrapolated to larger populations in different geographical regions and other dairies.

In addition to minimizing phenotyping variation, our GWAS used high-density SNP genotyping to increase the resolution of QTL detection. Previous studies [16,17,20,38,54] had larger sample sizes than our study, achieved by using dairies across multiple geographic regions and various lower-density SNP panels (maximum 76 K SNPs). The lower-resolution SNP panels in those studies may have prevented the detection of smaller linkage disequilibrium blocks (<20 kb) in Holstein cattle [55] and contributed to the inconsistency of genomic regions detected. Although two previous studies found associated loci on BTA1, for one study the suggestive SNPs were in a different region [6], while the other study did not provide SNP coordinates to permit comparisons [16]. Similarly, other GWASs also detected the associated SNPs on BTA3 [17,19] and BTA14 [6], but in different regions. Other GWASs did not detect SNPs on the same chromosomes as our GWAS [54] or did not detect any suggestive or significant SNPs [20]. The published GWASs with smaller sample sizes using the high-density SNP array were able to find SNPs associated for other traits in Holstein cattle, including digital cushion thickness [56], mastitis resistance [57], and fat deposition [58]. Our study using tightly controlled cases and controls was the first to use high-density SNP genotypes in a GWAS for DD susceptibility for improved resolution and the first to find significant and suggestive SNPs on BTA2, 7, 18, and 20 in regions containing likely candidate genes or genes in relevant pathways. The multiplicity of associated chromosomal regions supports that the genetic component of DD susceptibility is heterogeneous and highly complex, such that different combinations of loci with small effects contribute to DD susceptibility, as suggested by previous authors [20,38]. The complex genetic architecture of DD susceptibility likely reflects multiple physiological systems (e.g., immune system, hair morphology, skin matrix remodeling) interacting in the etiology of DD.

The lack of congruence in the genomic regions associated with DD across published studies and the small effect sizes of those QTLs identified further supports that, in addition to many low-impact loci, non-genetic factors strongly influence DD susceptibility. The ranking of farm as the most important predictor in the RF analyses supports the concept that farm management (e.g., hoof trimming regimen, methods of preventing and treating DD) plays a significant role in reducing DD prevalence. Employing genetic selection in combination with environmental management will likely further reduce DD prevalence.

## 5. Conclusions

GWAS using LMM and RF approaches identified loci containing six genes on BTA1, 7, and 20 that regulate skin integrity, immune function, and wound repair: *CXCR4*, *MGAT5*, *CACNA1A*, *TERT*, *SLC9A3*, and *AHRR*. Bayesian estimation of SNP effects was used to additionally distinguish between informative and noninformative SNPs and indicated that the top SNPs from LMM-binary and RF-quantitative were collectively predictive of binary and quantitative phenotypes. Despite our identifying significant QTL, the absence of the congruency of associated SNPs in this study compared to other studies and the consistent ranking of the farm as the most important predictor in the RF analyses support the notion that DD susceptibility is heavily influenced by management, and the remaining genetic component is heterogeneous and highly complex. Thus, although farm management may be the most effective short-term method for reducing DD prevalence, combining genetic selection with management will likely be the most effective and sustainable long-term solution.

## Figures and Tables

**Table 1:** Distribution of digital dermatitis cases and non-lame controls across the four dairies.

Farm	Case	Control	Total
A	19	112	131
B	22	0	22
C	30	0	30
D	16	17	33
Total	87	129	216

**Table 2:** Suggestive SNPs detected from the linear mixed model genome-wide association analysis using binary phenotypes and their defined QTL.

SNP ID	BTA	SNP Position (bp)	Minor/Major Allele	Minor Allele Count		MAF ^a^		Effect Size (SE)	*p*	Significance in Bayesian Regression	QTL Start Position (bp)	QTL End Position (bp)	QTL Size (kb)
				Cases (2n = 174)	Controls (2n = 258)	Cases	Controls						
BovineHD0100035768	1	125563251	A/G	63	54	0.362	0.209	0.178 (0.037)	1.68 × 10^−6^	ns	125550933 ^b^	125822143 ^b^	271.21 ^b^
BovineHD0100035771	1	125565548	G/A	63	54	0.362	0.211	0.175 (0.037)	2.31 × 10^−6^	ns	125550933 ^b^	125822143 ^b^	271.21 ^b^
BovineHD0100035773	1	125567245	T/C	63	55	0.362	0.213	0.175 (0.037)	2.35 × 10^−6^	ns	125550933 ^b^	125822143 ^b^	271.21 ^b^
BovineHD0100035776	1	125570173	G/T	63	55	0.362	0.213	0.175 (0.037)	2.35 × 10^−6^	ns	125550933 ^b^	125822143 ^b^	271.21 ^b^
BovineHD0100035780	1	125573042	G/A	63	55	0.362	0.213	0.175 (0.037)	2.35 × 10^−6^	ns	125550933 ^b^	125822143 ^b^	271.21 ^b^
BovineHD0100035783	1	125576193	G/A	63	55	0.362	0.213	0.175 (0.037)	2.35 × 10^−6^	ns	125550933 ^b^	125822143 ^b^	271.21 ^b^
BovineHD0100035788	1	125598084	G/A	63	57	0.362	0.221	0.164 (0.036)	5.36 × 10^−6^	ns	125550933 ^b^	125822143 ^b^	271.21 ^b^
BovineHD4100000712	1	125598643	T/C	63	57	0.362	0.223	0.163 (0.036)	6.31 × 10^−6^	ns	125550933 ^b^	125822143 ^b^	271.21 ^b^
BovineHD0100035789	1	125599413	C/T	63	57	0.362	0.221	0.164 (0.036)	5.36 × 10^−6^	ns	125550933 ^b^	125822143 ^b^	271.21 ^b^
BovineHD0100035796	1	125608174	A/G	64	56	0.368	0.217	0.163 (0.036)	4.59 × 10^−6^	ns	125550933 ^b^	125822143 ^b^	271.21 ^b^
ARS-BFGL-NGS-113021	1	125609019	C/T	64	56	0.368	0.217	0.163 (0.036)	4.59 × 10^−6^	ns	125550933 ^b^	125822143 ^b^	271.21 ^b^
BovineHD0100035797	1	125609959	C/T	64	56	0.368	0.217	0.163 (0.036)	4.59 × 10^−6^	ns	125550933 ^b^	125822143 ^b^	271.21 ^b^
BovineHD0100035802	1	125627579	C/T	64	56	0.368	0.217	0.163 (0.036)	4.59 × 10^−6^	ns	125550933 ^b^	125822143 ^b^	271.21 ^b^
BovineHD0100035803	1	125628401	A/C	64	56	0.368	0.217	0.163 (0.036)	4.59 × 10^−6^	ns	125550933 ^b^	125822143 ^b^	271.21 ^b^
BovineHD0100035828	1	125680990	G/A	87	88	0.500	0.341	0.155 (0.034)	4.11 × 10^−6^	ns	125550933 ^b^	125822143 ^b^	271.21 ^b^
BovineHD0100035829	1	125681850	C/A	87	88	0.500	0.341	0.155 (0.034)	4.11 × 10^−6^	ns	125550933 ^b^	125822143 ^b^	271.21 ^b^
ARS-BFGL-NGS-100109	1	125683184	C/T	87	88	0.500	0.341	0.155 (0.034)	4.11 × 10^−6^	ns	125550933 ^b^	125822143 ^b^	271.21 ^b^
BovineHD0100035833	1	125688941	C/T	87	88	0.500	0.341	0.155 (0.034)	4.11 × 10^−6^	ns	125550933 ^b^	125822143 ^b^	271.21 ^b^
BovineHD0100035841	1	125700410	A/G	87	89	0.500	0.345	0.152 (0.034)	5.85 × 10^−6^	ns	125550933 ^b^	125822143 ^b^	271.21 ^b^
BovineHD0100035842	1	125700857	C/T	87	89	0.500	0.345	0.152 (0.034)	5.85 × 10^−6^	ns	125550933 ^b^	125822143 ^b^	271.21 ^b^
BovineHD0100035844	1	125702010	C/T	87	89	0.500	0.345	0.152 (0.034)	5.85 × 10^−6^	ns	125550933 ^b^	125822143 ^b^	271.21 ^b^
BovineHD0100035845	1	125702906	G/T	87	89	0.500	0.345	0.152 (0.034)	5.85 × 10^−6^	ns	125550933 ^b^	125822143 ^b^	271.21 ^b^
BTA-47853-no-rs	2	63365256	A/G	78	64	0.448	0.248	0.167 (0.036)	3.69 × 10^−6^	s	60971364	63389576	2418.2
BovineHD0200019142	2	65836042	G/A	41	32	0.236	0.124	0.224 (0.046)	1.10 × 10^−6^	s	65836042	65836042	-

^a^ MAF = minor allele frequency. ^b^ This QTL is defined in both the linear mixed model and random forest analyses for the binary case-control phenotype. s = SNP effect estimated from Bayesian regression was significantly different from zero, as defined by the 95% uncertainty interval. ns = SNP effect estimated from Bayesian regression was not significantly different from zero, as defined by the 95% uncertainty interval.

**Table 3:** Important SNPs from random forest analysis using binary phenotypes and their defined QTL. Importance variables are expressed as % importance relative to farm (i.e., farm was 100% importance).

SNP ID	BTA	SNP Position (bp)	Minor/Major Allele	Minor Allele Count		MAF ^a^		SNP Importance (% Relative to Farm)	Significance in Bayesian Regression	QTL Start Position (bp)	QTL End Position (bp)	QTL Size (kb)
				Cases (2n = 174)	Controls (2n = 258)	Cases	Controls					
BovineHD0100001686	1	5894509	G/A	54	75	0.310	0.291	70.9	ns	5894509	5901795	7.3
BovineHD0100013452	1	47090630	C/T	41	25	0.238	0.098	75.4	ns	43459206	49409839	5950.6
BovineHD0100013140	1	45742004	G/A	35	20	0.201	0.078	75.6	ns	43459206	49409839	5950.6
BovineHD0100013551	1	47618749	T/G	50	27	0.291	0.105	81.5	ns	43459206	49409839	5950.6
BovineHD0100033878	1	118845470	A/G	9	41	0.052	0.159	76.1	ns	114235013	119003717	4768.7
BovineHD0100035876	1	125811728	A/C	70	68	0.402	0.264	79.4	s	125550933 ^b^	125822143 ^b^	271.21 ^b^
BovineHD0200017030	2	59626300	C/T	22	95	0.126	0.368	77.0	s	58016533	59967789	1951.3
BovineHD0200037724	2	129189118	T/C	35	73	0.201	0.283	83.0	ns	128495987	129671807	1175.8
BovineHD0300035231	3	119898047	T/G	52	53	0.299	0.205	76.7	s	119720909	119942789	221.9
BovineHD0400033808	4	115632631	A/G	84	92	0.483	0.357	76.5	ns	115461900	115812750	350.9
ARS-BFGL-NGS-111175	4	119082548	A/C	38	30	0.218	0.116	76.8	ns	116927673	119130213	2202.5
BovineHD0400034694	4	117654227	G/A	53	119	0.305	0.461	76.9	ns	116927673	119130213	2202.5
BovineHD0700005793	7	19675119	C/T	87	102	0.500	0.395	75.5	ns	17910021	19773720	1863.7
BovineHD0700016221	7	54331048	A/G	6	42	0.034	0.163	77.0	ns	49401649	54505899	5104.3
BovineHD1300007641	13	26082265	C/T	69	140	0.397	0.543	76.0	ns	22185154	26101077	3915.9
BovineHD1500016894	15	57724182	A/G	60	59	0.345	0.229	72.9	ns	56807906	58102169	1294.3
BovineHD1600016687	16	58237523	C/T	105	108	0.603	0.419	81.8	ns	56372228	62230342	5858.1
BovineHD1700012893	17	45209840	T/C	62	52	0.356	0.202	80.4	ns	44418753	45224548	805.8
BovineHD1800003369	18	9579005	T/C	100	102	0.575	0.395	79.6	ns	9510127	9582839	72.7
BovineHD1800012376	18	41782168	C/T	27	8	0.155	0.031	88.4	ns	41753915	41863187	109.3
ARS-BFGL-BAC-35025	18	47814171	G/A	32	84	0.184	0.326	79.8	s	47099464	47831459	732.0
BovineHD1900013252	19	46915144	C/T	27	90	0.155	0.349	86.2	ns	46871178	47070613	199.4
BovineHD2200002436	22	8104318	A/G	36	106	0.207	0.411	79.8	ns	7974675	8109630	135.0
BovineHD2200002746	22	9090720	A/G	17	77	0.098	0.298	85.0	ns	9068141	9090720	22.6
BovineHD2600011849	26	42398008	A/G	59	68	0.339	0.264	75.6	ns	40792161	43877138	3085.0
ARS-BFGL-NGS-117055	27	12656552	C/T	86	89	0.494	0.348	75.7	ns	12202138	12834272	632.1

^a^ MAF = minor allele frequency. ^b^ This QTL is defined in both the linear mixed model and random forest analyses for the binary case-control phenotype. s = SNP effect estimated from Bayesian regression was significantly different from zero, as defined by the 95% uncertainty interval. ns = SNP effect estimated from Bayesian regression was not significantly different from zero, as defined by the 95% uncertainty interval.

**Table 4:** Significant and suggestive SNPs detected from the linear mixed model genome-wide association analysis using quantitative phenotypes and their defined QTL.

SNP ID	BTA	SNP Position (bp)	MAF ^a^	Effect Size (SE)	*p*	QTL Start Position (bp)	QTL End Position (bp)	QTL Size (kb)
BovineHD0200022555	2	78069923	0.231	0.127 (0.025)	3.14 × 10^−7^ *	77930065	79925981	1995.9
BovineHD0200022557	2	78080217	0.231	0.127 (0.025)	3.14 × 10^−7^ *	77930065	79925981	1995.9
Hapmap43777-BTA-115985	2	78080944	0.233	0.128 (0.025)	2.66 × 10^−7^ *	77930065	79925981	1995.9
BovineHD0200022559	2	78092854	0.231	0.127 (0.025)	3.14 × 10^−7^ *	77930065	79925981	1995.9
BovineHD0200022560	2	78100071	0.231	0.127 (0.025)	3.14 × 10^−7^ *	77930065	79925981	1995.9
BovineHD0200022562	2	78110140	0.231	0.127 (0.025)	3.14 × 10^−7^ *	77930065	79925981	1995.9
BovineHD0200022563	2	78111523	0.231	0.127 (0.025)	3.14 × 10^−7^ *	77930065	79925981	1995.9
BovineHD0200022605	2	78307821	0.28	0.107 (0.023)	3.68 × 10^−7^ †	77930065	79925981	1995.9
BovineHD0200022737	2	78767889	0.278	0.108 (0.023)	3.43 × 10^−7^ †	77930065	79925981	1995.9

^a^ MAF = minor allele frequency. * = genome-wide significant. † = genome-wide suggestive significance.

**Table 5:** Important SNPs from random forest analysis using quantitative phenotypes and their defined QTL. Importance variables are expressed as % importance relative to farm (i.e., farm had 100% importance).

SNP ID	BTA	SNP Position (bp)	Minor/Major Allele	MAF ^a^	SNP Importance (% Relative to Farm)	Significance in Bayesian Regression	QTL Start Position (bp)	QTL End Position (bp)	QTL Size (kb)
BovineHD0100036283	1	127408427	A/G	0.350	8.9	ns	127389567	127408427	18.9
BovineHD0300023756	3	82473975	A/G	0.391	8.5	ns	82468446	82480613	12.2
BovineHD0700003488	7	12238249	T/G	0.354	17.1	s	11979738	12261707	282.0
BovineHD0700023293	7	77533459	T/C	0.220	8.7	ns	77242189	78032023	789.8
BovineHD0800002826	8	8983282	C/T	0.463	8.7	ns	8671707	9806692	1135.0
BovineHD0800002824	8	8979816	G/A	0.373	10.6	ns	8671707	9806692	1135.0
BovineHD0800030529	8	100994105	C/T	0.402	10.5	ns	100412296	102353854	1941.6
BovineHD0800030627	8	101328029	G/A	0.350	9.5	ns	100412296	102353854	1941.6
BovineHD1100025931	11	89788438	C/A	0.387	9.9	ns	89375874	89788438	412.6
BovineHD1400011939	14	39785964	T/C	0.448	10.0	s	39785964	39818361	32.4
BovineHD1500006588	15	24668401	A/G	0.250	11.2	ns	24668401	24771237	102.8
UA-IFASA-9742	15	42081374	G/T	0.250	8.6	ns	42081374	42092689	11.3
BovineHD1800012376	18	41782168	C/T	0.081	9.6	ns	41753915	41863187	109.3
BovineHD2000020460	20	69870827	T/C	0.308	8.3	s	69696705	71850045	2153.3
BovineHD2200002433	22	8091674	T/C	0.205	9.1	ns	6375507	8317371	1941.9

^a^ MAF = minor allele frequency. s = SNP effect estimated from Bayesian regression was significantly different from zero, as defined by the 95% uncertainty interval. ns = SNP effect estimated from Bayesian regression was not significantly different from zero, as defined by the 95% uncertainty interval.

**Table 6:** Quantitative trait loci (QTL) defined by SNPs that were significant in at least two analyses: linear mixed model (LMM), random forest (RF), or Bayesian regression of top SNPs from linear mixed model (LMM-B) or random forest (RF-B) containing functionally relevant pathways or genes.

Phenotype	BTA	QTL Start Position (bp)	QTL End Position (bp)	QTL Size (kb)	Methodology Used in Defining the QTL	Relevant Pathways	Candidate Genes in QTL
Binary	1	125550933	125822143	271.2	LMM, LMM-B, RF, RF-B		
	2	60971364	63389576	2418.2	LMM, LMM-B		*CXCR*, *MGAT5*
	2	58016533	59967789	1951.3	RF, RF-B		
	2	65836042	65836042	-	LMM, LMM-B		
	3	119720909	119942789	221.9	RF, RF-B		
	18	47099464	47831459	732.0	RF, RF-B	Herpes simplex virus 1 infection	
Quantitative	7	11979738	12261707	282.0	RF, RF-B		*CACNA1A*
	14	39785964	39818361	32.4	RF, RF-B		
	20	69696705	71850045	2153.3	RF, RF-B		*TERT*, *SLC9A3*, *AHRR*

## Supplementary Materials

The following are available online at https://www.mdpi.com/2076-2615/10/11/2009/s1: Table S1. Suggestive SNPs detected from the linear mixed model genome-wide association analysis using recurrent phenotypes and their defined QTL. Table S2. Candidate genes found within the nine QTL defined by SNPs that were significant/important in at least two of the following analyses: linear mixed model, random forest, and/or Bayesian regression. Figure S1. Multidimensional scaling plot depicting the first two dimensions. Each dot represents a cow, status is indicated by point shape, and farm is indicated by point color. Figure S2. Manhattan plot for the linear mixed model genome-wide association analysis using binary phenotypes from recurrent cases vs. controls. Figure S3. Quantile-quantile plots depicting observed and expected p-values from linear mixed model genome-wide association analyses using (a) binary, (b) quantitative, and (c) binary recurrent phenotypes in the full dataset of 261 cows; and (d) binary and (e) quantitative phenotypes in the subset of 188 cows after removing outlier cows. The red line indicates when observed and expected *p*-values are equivalent. Figure S4. Manhattan plots from linear mixed model genome-wide association analyses excluding the outlier control cows using (a) binary phenotypes designating the presence of digital dermatitis (DD) lesions or absence of any lameness issues and (b) quantitative phenotypes calculated by dividing the number of DD episodes by the total number of hoof trimming records. The red line indicates the threshold for genome-wide significance (Bonferroni-corrected using the number of independent SNPs at *p* < 0.05), and the blue line indicates the threshold for suggestive significance (Bonferroni-corrected using the number of independent SNPs at *p* < 1). Genomic inflation factors (lambda) are indicated in figure titles. Figure S5. Posterior predictive check bar plot for Bayesian regression estimating effects of suggestive SNPs detected in the linear mixed model genome-wide association study using binary phenotypes. Gray bars represent the actual phenotypes and black dots with intervals represent the median and uncertainty intervals of the phenotypes of replicates (y_rep_), which were simulated from estimated effects of predictors. Figure S6. Posterior predictive check bar plot for Bayesian regression estimating effects of suggestive SNPs detected in the random forest using binary phenotypes. Gray bars represent the actual phenotypes and black dots with intervals represent the median and uncertainty intervals of the phenotypes of replicates (y_rep_), which were simulated from estimated effects of predictors. Figure S7. Posterior predictive check distribution plot for Bayesian regression estimating effects of suggestive SNPs detected in the random forest using quantitative phenotypes. The black line represents the actual phenotypic distribution and grey lines dots represent the phenotypic distribution of replicates (y_rep_), which were simulated from estimated effects of predictors.
